# Metabolic Gadolinium Labeling of *Clostridium novyi*-Nontoxic for Magnetic Resonance Imaging Visualization of Spores and Germinated Bacteria

**DOI:** 10.34133/bmr.0326

**Published:** 2026-02-10

**Authors:** Hyunjun Choi, Jun-Hyeok Han, Sanghee Lee, Dong-Hyun Kim

**Affiliations:** ^1^ Department of Radiology, Feinberg School of Medicine, Northwestern University, Chicago, IL 60611, USA.; ^2^ Department of Biomedical Engineering, McCormick School of Engineering, Evanston, IL 60208, USA.; ^3^ Robert H. Lurie Comprehensive Cancer Center, Chicago, IL 60611, USA.; ^4^ Department of Biomedical Engineering, University of Illinois at Chicago, Chicago, IL 60607, USA.

## Abstract

Bacterial cancer therapy using *Clostridium novyi-*nontoxic (*C. novyi-*NT) has emerged as a promising strategy to selectively eradicate hypoxic and necrotic tumor regions. However, noninvasive visualization of injected spores and germinated bacteria remains a major barrier to clinical translation. Here, we developed metabolically responsive gadolinium-labeled *C. novyi-*NT (Gd-*C. novyi-*NT) to enable magnetic resonance imaging (MRI)-based tracking of spore localization and bacterial germination. Gd ions were electrostatically condensed onto the negatively charged spore surface, forming Gd clusters that produced strong T2-weighted contrast without affecting spore viability or germination efficiency. Under anaerobic conditions, these Gd aggregates were progressively diluted and redistributed along the bacterial cell wall through metal-chelating metabolites, converting the MRI signal from T2-dominant to T1-weighted contrast. In an orthotopic liver tumor model, intra-arterial infusion of Gd-spores led to selective intratumoral accumulation, with early T2 contrast followed by sustained T1 enhancement persisting for up to 9 days post-administration. This metabolically driven T2-to-T1 contrast transition provides a dynamic, noninvasive imaging signature that correlates with bacterial germination and proliferation. The Gd-*C. novyi-*NT platform therefore offers a powerful tool for real-time monitoring of bacterial therapeutic behavior in vivo, advancing precision imaging and therapeutic control of bacterial-based oncologic therapies.

## Introduction

Cancer remains a leading cause of mortality worldwide [1]. Despite substantial progress in conventional therapies, including chemotherapy, radiotherapy, and immunotherapy, many malignancies remain challenging to treat [2]. The lack of tumor-specific targeting, together with the intrinsic therapeutic resistance associated with severe intratumoral hypoxia, represents a major limitation of current cancer treatments [3]. Thus, diverse strategies have been developed to improve tumor selectivity and overcome hypoxia-driven resistance mechanisms [4]. Notably, the hypoxic and necrotic tumor microenvironment that undermines conventional therapies provides an ideal niche for the growth of obligate anaerobic bacteria [5,6]. Although clostridia are strictly anaerobic, they possess the unique ability to form dormant spores, enabling survival, but not proliferation, under oxygen-rich conditions [7]. These spores only germinate into metabolically active cells when conditions are favorable. Bacterial cancer therapy has emerged as a promising strategy to specifically target and eradicate therapeutically resistant tumors by selectively colonized bacteria in the hypoxic and necrotic regions of tumors [8,9]. Furthermore, the oncolytic effect of bacteria can activate an antitumor immune response, acting as a natural adjuvant to enhance the body’s ability to recognize and destroy cancer cells [10–12].

*Clostridium novyi*-nontoxic (*C. novyi*-NT), which has been genetically modified to lack the lethal alpha-toxin, has gained marked attention in bacterial cancer therapy by its ability to selectively thrive in hypoxic tumor environments due to its obligatory anaerobic growing conditions that provide precise targeting of bacterial growth in the tumor [13]. Unlike other bacterial strains typically used for therapeutic purposes, *C. novyi*-NT is administered in its spore form. However, the intratumoral germination of *C. novyi*-NT varies substantially depending on the tumor microenvironment and the patient’s health conditions [13–15]. This interindividual variability in the germination *C. novyi*-NT has been found to have a substantial impact on the efficiency of cancer treatment. Although invasive procedures such as blood sampling and biopsies have been the options to investigate the nonspecific germination in such patients, those results do not directly monitor the tumoral distribution of injected *C. novyi*-NT spores and their germination in the tumor or other tissues. Noninvasive visualization and tracking of bacteria within tumors are critically beneficial for the successful application and development of bacteria-based cancer therapies [16,17]. Further, monitoring the proliferation of therapeutic bacteria inside the tumor microenvironment also provides valuable insights into treatment efficacy, safety, and mechanisms of action [18]. Thus, various labeling and imaging technologies such as bioluminescence imaging, fluorescence imaging, photoacoustic imaging, positron emission tomography, and Raman imaging have been studied for real-time visualization of bacterial distribution, proliferation, and interaction with the tumor microenvironment [19–21]. However, insufficient imaging depth and limited detectable time have been the issues for the translation of proposed imaging methods. Magnetic resonance imaging (MRI) offers deep tissue imaging with high spatial resolution, making it an excellent tool for tracking bacterial migration and distribution in tumors. MRI visible bacteria labeling methods using superparamagnetic iron oxide nanoparticles, ultrasmall superparamagnetic iron oxide nanoparticles, and gadolinium (Gd)-chelated conjugates are some of the most promising approaches for clinical uses [22,23]. Intrinsic magnetic labeling such as magnetotactic bacteria having iron oxide crystals has been suggested for long-term MRI monitoring, but there are very limited options in the sensitivity and strain and genetic engineering complexity with regulatory concerns [24–26]. Exogenous magnetic labeling of bacteria has been proposed as a promising approach, leveraging established superparamagnetic iron oxide nanoparticles as T2 contrast agents and paramagnetic Gd-chelated conjugates as T1 contrast agents [27,28]. Their high sensitivity is advantageous for detecting and monitoring bacteria during bacterial cancer therapy procedure. The labeling process is typically achieved through covalent and electrostatic interactions. However, maintaining labeling stability and reliable MR signal readouts becomes challenging as spores undergo dynamic metabolic transitions during germination and bacterial outgrowth. Most prior studies have demonstrated that magnetic nanoparticles are effective for tracking spores before germination [27,29]. In contrast, longitudinal MR monitoring after germination is often incomplete or lost, unless genetic engineering strategies are employed [30,31].

**Fig. 1.**
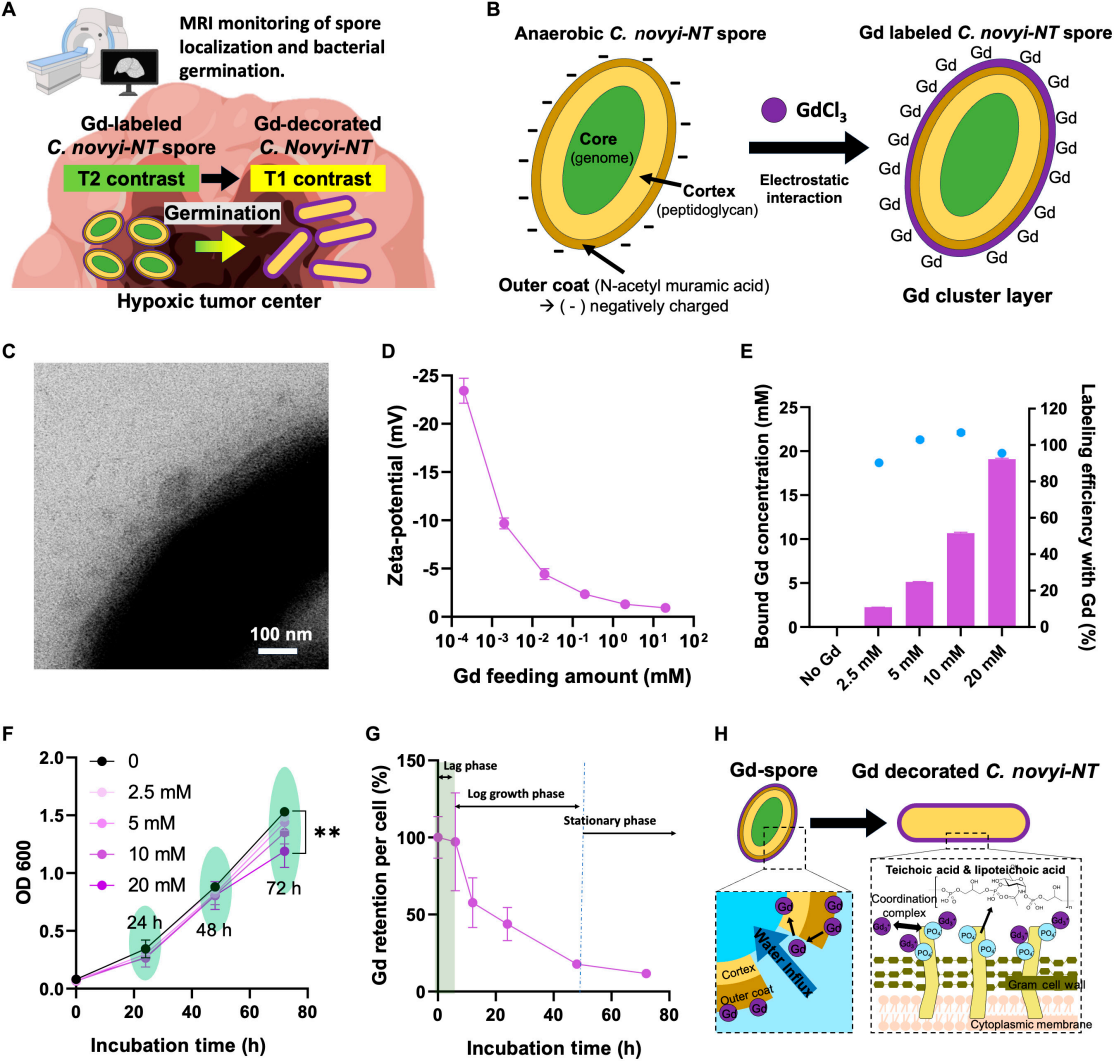
Characterization of gadolinium-labeled *C. novyi-*NT spores (Gd-spores). (A) Schematic illustration of the metabolic MRI contrast transition of Gd-labeled spores during germination under hypoxia. (B) Schematic of the electrostatic coordination of Gd^3+^ ions onto the negatively charged spore coat and cortex. (C) Transmission electron microscopy (TEM) image confirms the formation of Gd cluster layer on spore cell walls. (D) Zeta potential analysis showing surface charge neutralization of spores upon GdCl_3_ addition (*n* = 3). (E) Gd loading amount (purple bars) and Gd loading efficiency (blue dots) in spores as a function of GdCl_3_ feeding concentration (*n* = 3). (F) Growth curve (OD_600_) of *C. novyi-*NT spores incubated with different Gd concentrations (0 to 20 mM) (*n* = 3). (G) Time-dependent retention of Gd content during germination of Gd-spores (10^8^/ml, prepared with 10 mM GdCl_3_) (*n* = 3). (H) Proposed schematic of Gd redistribution from spores to vegetative bacterial membranes via phosphate coordination with teichoic acids. Data are presented as mean ± SD (**P < 0.01).

Here, we introduce Gd-labeled *C. novyi*-NT spores (Gd-spores) that exhibit metabolically responsive MRI T2/T1 contrast conversion, enabling noninvasive monitoring of both localized *C. novyi*-NT spores delivery and subsequent bacterial germination within tumors (Fig. 1A). The feeding amounts and labeled amounts of Gd ions bound on *C. novyi*-NT were systemically optimized, and bacterial viability and proliferation were confirmed to be preserved within a safe Gd dosage window. An optimized Gd labeling of *C. novyi*-NT spores demonstrated strong T2-weighted signal with T1 signal void, attributable to paramagnetic susceptibility effect arising from densely clustered surface bound Gd. Upon germination, secretion of rare-earth-chelating metabolites by *C. novyi*-NT dynamically redistributed and diluted these Gd clusters, converting the initial T2-dominant signal into T1-weighted contrast enhancement and enabling real-time MRI visualization of bacterial germination. In vitro MRI phantom studies demonstrated robust tracking of both spores and germinated bacteria, including bacterial activity and migration. Finally, in vivo MRI studies in an orthotopic N1S1 rat hepatoma model validated this metabolically driven T2-to-T1 contrast transition, confirming the feasibility of noninvasive, longitudinal monitoring of spore localization and bacterial proliferation.

## Materials and Methods

### Materials

*C. novyi-*NT was kindly provided by BioMed Valley Discoveries (Kansas City, MO, USA). Gadolinium (III) chloride hexahydrate were purchased from Sigma-Aldrich (St Louis, MO, USA). *C. novyi* spore media was prepared with 5 g of Na_2_HPO_4_, 30 g of peptone, 0.5 g of L-cysteine, 10 g of maltose, and 5 w/v% dried cooked meat particles (DIFCO) in 1 l of distilled water. *C. novyi* culture media was prepared with RCM broth supplemented with 10% EC oxyrase to support anaerobic growth. The media was autoclaved at 121 °C for 15 min and stored at 4 °C until use. Other reagents and materials were used for further experiments without purification.

### Gd labeling of spores and bacteria

To evaluate the possible proliferative effect by the co-incubation with gadolinium on *C. novyi-NT* spores and vegetative bacteria, Gd-spores (10^8^/ml) prepared with varying concentrations of gadolinium (0 to 20 mM) were incubated in clostridium media under hypoxic conditions for up to 72 h. The samples were subsequently subjected to dialysis using membranes with a molecular weight cutoff of 10 kDa in sodium acetate buffer to remove excess gadolinium. Surface charge alterations were analysis by zeta potential measurements (Zetasizer Nano ZS, Malvern). Gd content was quantified by inductively coupled plasma mass spectrometry (ICP-MS; iCAP Q, Thermo Fisher). Morphology integrity and surface coating of Gd-spores were further examined by scanning electron microscopy with energy-dispersive x-ray spectroscopy (SEM-EDS; JEOL Neoscope) and transmission electron microscopy (TEM; FEI Spirit G2), confirming preserved cell wall structures and the formation of a Gd-clusters layer on the spore surface.

### Gd-labeled bacterial viability

Gd-spores were cultured in culture medium at an initial optical density of 0.1 at 600 nm (OD_600_ = 0.1) and incubated under hypoxic conditions for up to 72 h at 37 °C. Hypoxia was established using an anaerobic GasPak EZ system (BD Becton Dickinson) placed in sealed incubation jars, which generated a microaerophilic to anaerobic atmosphere within 30 min of activation. Bacterial growth and viability were monitored by measuring OD_600_ at 0, 24, 48, and 72 h using a microplate reader (Cytation 3, Biotek). Each measurement was performed in triplicate to ensure reproducibility.

### In vitro MRI monitoring of Gd-spores and Gd-bacteria

Agar phantoms (0.1% agar) were prepared in 1.5-ml Eppendorf tubes. Gd-spores (10 mM Gd in 10^8^ spores) or unlabeled controls were injected into phantom cores and incubated under hypoxic or normoxic conditions. In parallel, to evaluate the proliferative migration of Gd-labeled *C. novyi-*NT, 35-mm culture dishes were filled with 0.1% agar, and a 5-mm central sample hole was created in each dish. Gd-spores (10 mM Gd in 10^8^ spores) or unlabeled controls were injected into sample hole and incubated under hypoxic or normoxic conditions. Time-dependent MRI was performed on a 7-T MRI scanner (Bruker BioSpin, Ettlingen) using T1- and T2-weighted spin-echo sequences. Regions of interest were defined at injection point, boundary, and tube tip. Relaxation times were calculated using Paravision 6.0 software.

### In vivo hepatic arterial infused Gd-spores and MRI monitoring

All experimental procedures were approved and reviewed by the Institutional Animal Care and Use Committee of Northwestern University (IS00003865) and followed procedures described in our previous study [16,18]. N1S1 rat hepatoma cells (ATCC CRL-1603) were cultured with Iscove’s Modified Dulbecco’s Medium, and 3 × 10^6^ cells in 100 μl of phosphate-buffered saline (PBS) were inoculated in the left lateral lobe of the liver region of rats after the laparotomy. Tumor development was monitored for 7 days post-inoculation using a T2-weighted turbo spin-echo sequence on a 9.4-T Bruker Biospec MRI system (Bruker Biospin, Ettlingen, Germany) (TR/TE = 1,053/30 ms, turbo-factor = 12, 256 × 256 matrix, 130 × 130 mm field of view, 30 slices at 2 mm thickness, 200 Hz/px bandwidth). Once the tumor diameter reached 4 mm, intra-arterial (IA) delivery of Gd-spores was performed under isoflurane anesthesia (3%) by catheterization, and 100 μl of Gd-spores suspended in PBS was slowly infused into the proper hepatic artery. The catheter entry site was ligated, and normal hepatic blood flow was restored. After the injection, the hole made by catheterization was treated with silk sutures, and the bulldog clamp on the common hepatic artery was gently removed. After checking the bleeding on the blood vessel treated, the incision closure procedure was performed on the muscle and skin layer. Post-treatment tumor progression was monitored by MRI for up to 9 days. MRI data were analyzed using RadiAnt DICOM Viewer software.

### Statistical analysis

The results were expressed as the mean ± SD, and GraphPad Prism Version 8 (GraphPad Software) was utilized for the statistical data analysis. Statistical analyses were performed using one-way analysis of variance for obtaining *P* values. The *P* value was denoted by * for *P* < 0.05, ** for *P* < 0.01, *** for *P* < 0.001, and **** for *P* < 0.0001.

## Results and Discussion

### Gd labeling of *C. novyi*-NT spores (Gd-spores)

*C. novyi*-NT spores consist of 3 major regions: the core containing the genome, the intermediate cortex, and the protective outer coat layer. The outer coat comprises a protein-rich surface containing N-acetyl muramic acid, resulting in a negatively charged surface [32]. This negative charge in spores is primarily attributed to the presence of acidic functional groups, such as carboxyl and phosphate groups, on their surface proteins and polysaccharides. These groups contribute to the overall negative surface charge, which influences interactions with their environment, including adhesion properties and stability in suspension. The anionic surface of the outer membrane was utilized for labeling gadolinium (Gd^3+^) cations via electrostatic binding (Fig. 1B). The addition of gadolinium chloride (GdCl_3_) solutions (0 to 20 mM) to a suspension containing 10^8^ *C. novyi*-NT spores generated Gd-labeled *C. novyi*-NT spores (Gd-spores). A Gd cluster-coated layer was detected on the spore in TEM images (Fig. 1C). Consistently, SEM–EDS elemental mapping further confirmed the presence and surface localization of Gd on the spores (Fig. S1). The electrostatic binding between spores and Gd^3+^ was also confirmed with the zetapotential changes of spores upon the addition of GdCl_3_ (0 to 20 mM) (Fig. 1D). The negative surface charge (−26 mV) of spores was gradually increased to neutral surface charge by the addition of GdCl_3_. When 10 mM GdCl_3_ was added to the 10^8^ spores, the surface charge of Gd-labeled spores was saturated. Actual Gd amounts labeled in the spores were measured with ICP elemental analysis. The Gd labeling efficiency of 10^8^ spores was 90.3% to 100% in the addition of GdCl_3_ (0 to 20 mM). Under these Gd feeding conditions, the amount of Gd bound to the spores could be controlled by increasing the GdCl₃ feeding concentration (Fig. 1E). Potential toxicity arising from Gd labeling process is a critical consideration for preserving the oncolytic efficacy of *C. novyi*-NT spores. Accordingly, time-dependent proliferation of spores labeled with varying Gd amounts was assessed by monitoring OD_600_ (Fig. 1F). Across the tested Gd feeding concentration range, no significant suppression of *C. novyi*-NT spores’ proliferation was observed over a 72-h incubation period. Because a modest reduction in spore proliferation was observed in the 20 mM Gd treatment group, Gd-*C. novyi*-NT spores labeled using 10 mM GdCl₃ were selected for all subsequent studies. Overall, the gadolinium labeling strategy effectively modified *C. novyi*-NT spores while preserving their viability and capacity for germination.

### Metabolic Gd labeling of vegetative *C. novyi*-NT bacteria from Gd-spore

The exogeneous Gd cluster amount labeled on the outer membrane of spores was changed over time during their germination and division process. Here, the proliferation time-dependent Gd amounts change in a spore, or bacteria was quantified with an elemental analysis using ICP (Fig. 1G). The labeled Gd amounts per *C. novyi-*NT were initially maintained with only 2% decrease for 5 h, as the period corresponds to the lag phase of *C. novyi*-NT proliferative growth curve. However, following the lag phase between 5 and 10 h incubation, the labeled Gd amount decreased markedly from 98% to 58% as a result of proliferative binary divisions. As further incubation is going on with the exponential growth phase and stationary phase, the Gd retention rate was gradually decreased down to 20% in 72 h. However, the total Gd amount labeled in spores was maintained during the proliferative process. The resulting labeled Gd amount change in a *C. novyi-*NT is well matched with the proliferation curve as shown in Fig. 1F. This demonstrated that the initially labeled Gd was transferred to the germinated bacterial cell wall and the Gd amounts in bacteria were diluted throughout the metabolic proliferative processes. Gd transferring from spores to germinated bacteria is explained by metabolically generating bacterial cell walls (Fig. 1H). The forming bacterial cell walls from the initial spores are enriched in teichoic acids (TAs) and lipoteichoic acids (LTAs), which efficiently chelate Gd ions originally bound to the spore surface [33,34]. Bacterial cell wall biogenesis initiates with the disruption of the spore shell, followed by rapid water influx and hydration of the spore core. This hydration process increases the permeability of the inner membrane, permitting the transport of small molecules. Concurrently, de novo peptidoglycan synthesis begins, reconstructing the rigid bacterial cell wall required for vegetative growth. The newly formed bacterial cell wall is enriched with anionic polymers, including TAs and LTAs, which contain abundant phosphate and carboxylate groups that confer a high negative charge density. These phosphate moieties exhibit strong coordination affinity toward metal cations, particularly trivalent Gd^3+^ ions, enabling effective chelation and stable coordination of gadolinium (Gd^3+^) along the bacterial cell wall.

### Metabolic responsive MRI T2 and T1 contrast shift of Gd-spore

**Fig. 2.**
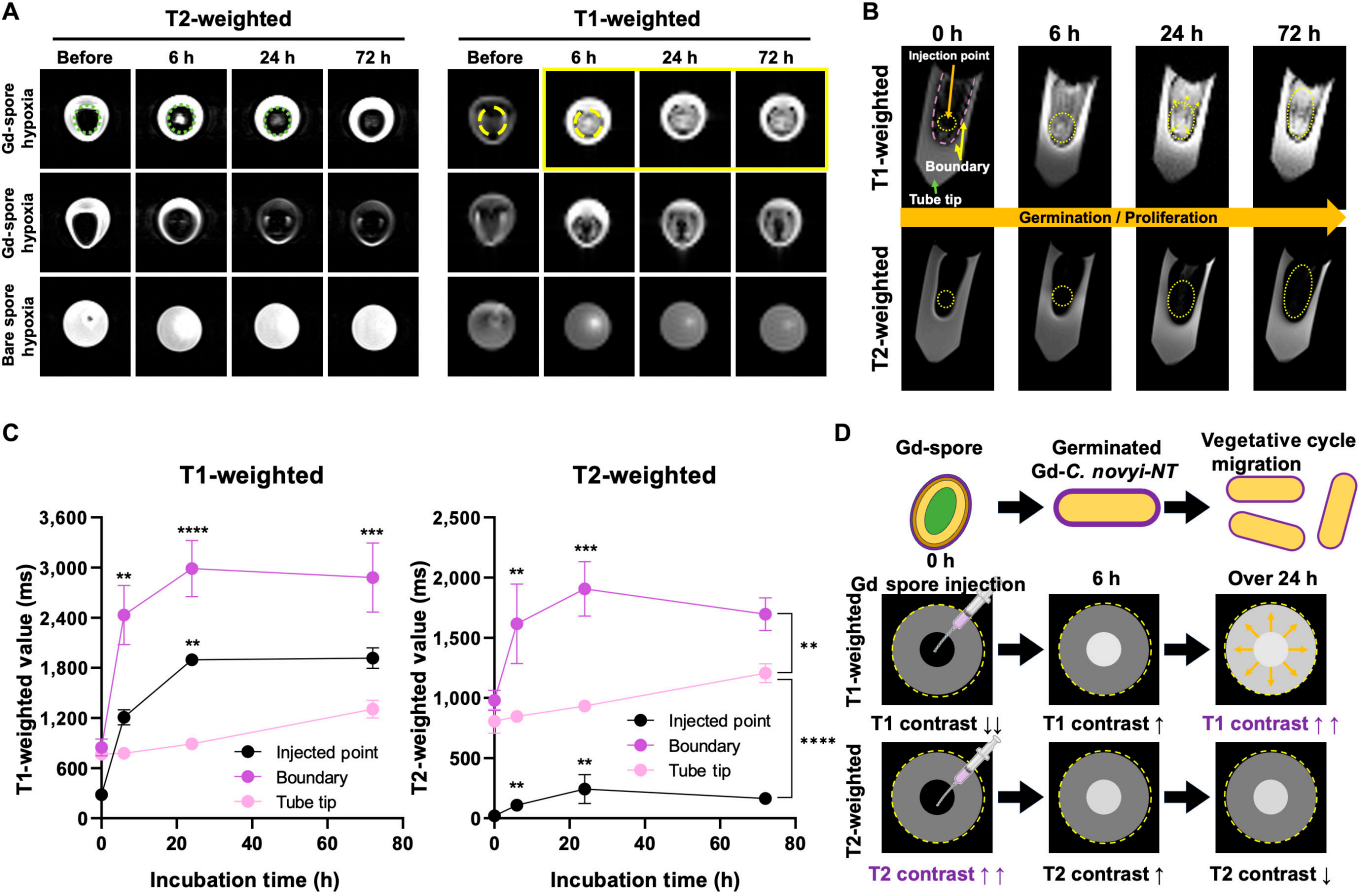
MRI phantom analysis of Gd-spores during germination. (A) Transverse MR images of agar phantom tubes containing Gd-spores (10^8^/ml, prepared with 10 mM GdCl_3_) incubated under metabolic proliferative hypoxia or nonproliferative normoxia. (B) Vertical MR images showing spatial distribution of T1 and T2 contrast at Gd-spore injection points. (C) Quantitative T1 and T2 relaxation times measured at the injection point, the boundary region, and the tube tip (*n* = 3). (D) Schematic illustration of metabolic T2-to-T1 transition enabling visualization of Gd-spore injection, germination, and proliferation. Data are presented as mean ± SD (**P < 0.01, ***P < 0.001, ****P < 0.0001).

Temporal changes of Gd amount labeled in spores and germinated bacterial are critical for understanding the MRI visibility of Gd-*C. novyi*-NT at defined time points. Accordingly, the metabolism-dependent MRI contrast effect of Gd-spores was evaluated using T2- and T1-weighted MRI following localized injection of Gd-*C. novyi*-NT spores (Gd: 10 mM in 10^8^ spores) into an agar phantom fabricated with *Clostridium* growth media. As shown in Fig. 2A, Gd-spores were injected in the local area to mimic intratumoral injection and incubated for up to 72 h under either metabolically permissive hypoxic conditions or nonproliferative normoxic conditions. Injected Gd-spores exhibited a pronounced T2-weighted contrast with a corresponding T1 signal void in transverse MR images of the sample tubes, whereas non-Gd-labeled spores showed no appreciable T1 or T2 contrast. After 6-h incubation under hypoxia, a strong T1 contrast effect emerged, consistent with bacterial germination and metabolic activity. In contrast, samples maintained under normoxic, nonproliferative conditions exhibited no appreciable changes in either T2 or T1 contrast. To further resolve spatial contrast evolution, vertical MRI scans of the Gd-spore-injected agar phantoms were subsequently acquired (Fig. 2B). When the Gd-spores were injected, a concentrated Gd (10 mM) on the surface of spores exhibited a strong T2 contrast effect (11 ms) rather than T1 (220 ms) in the injection point area (yellow circles) (Fig. 2B and C). Gd-contrast agents are primarily used to enhance T1-weighted MRI signals. However, a high Gd concentration (~10 mM) on the surface of spores demonstrated T1 void effects with the dominance of T2 contrast effects increasing the dephasing effect [35–37]. Gd ions exhibit markedly higher magnetic susceptibility than water and biological tissues. When present at high local concentrations, Gd induces strong local field inhomogeneities (microscopic variations in magnetic field strength around the ions) that accelerate proton spin dephasing and resulting in pronounced signal attenuation/darkening on T2-weighted images. This MRI contrast behavior was inversely transitioned from T2 to T1 dominance as a result of metabolic dilution and redistribution of Gd on the bacterial outer membranes. After 6-h hypoxia incubation, pronounced T1-weighted signal enhancement (1,200 ms) was observed at the injection area, accompanied by residual T2 contrast (150 ms). As *C. novyi*-NT metabolic fissions increase with the extended incubation time by 72 h, T1 signal increased up to 1,950 ms at 24 h and the saturated value was maintained in 72 h of incubation. T2 contrast effect was slightly increased in the early stage of proliferation, but the T2 signal eventually decreased in 72 h of incubation. These confirm the T1 dominance of Gd-*C. novyi*-NT bacteria with the metabolic growth of bacteria with the dilution of Gd concentration on the surface of spores and germinated bacteria. Additionally, time-dependent migration of vegetative bacteria can be monitored with spatial T1 contrast changes in the tube sample. T1 contrast of Gd-*C. novyi*-NT in the boundary area increased from 800 ms to a maximum of 3,000 ms for 24 h incubation in a metabolic hypoxia condition (Fig. 2C). At the same time, T1 signal at the injection point is also increased from 220 ms to a maximum of 1,900 ms. These signal changes demonstrate the metabolic migration of germinated Gd-*C. novyi*-NT bacteria. Taken together, the demonstrated dynamic metabolic responsive MRI T2/T1 transition of Gd-*C. novyi*-NT will allow the tracking of the initial delivery of Gd-spores in MRI T2 scanning and distribution of vegetative Gd-bacteria via sequential T1 MRI (Fig. 2D).

### MRI monitoring of Gd-labeled *C. novyi*-NT proliferative migration

**Fig. 3.**
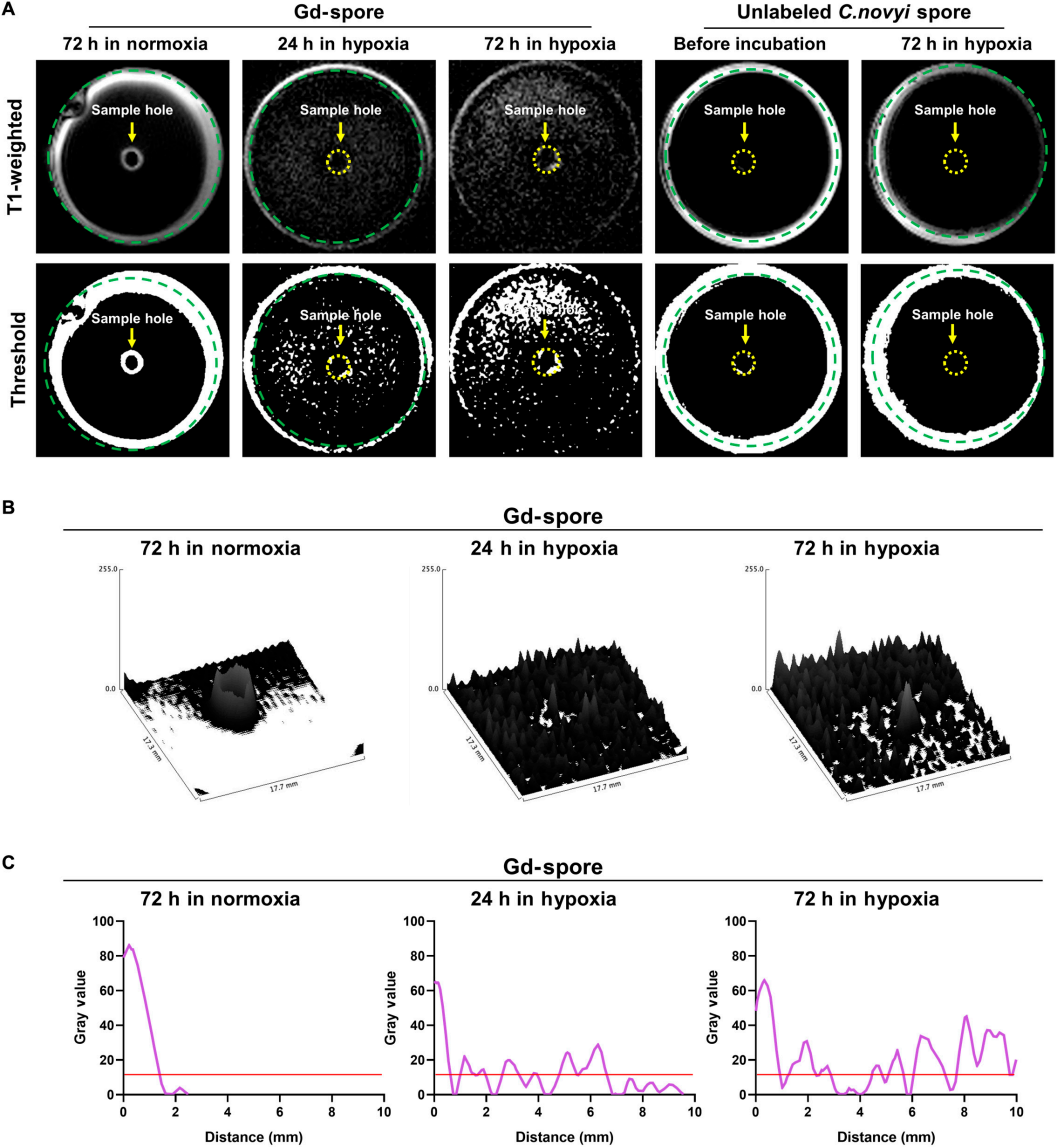
Monitoring T1 signal propagation of Gd-spores in agar phantom. (A) Comparison of T1 signal changes between Gd-spores (10^8^/ml, prepared with 10 mM GdCl_3_) and unlabeled *C. novyi-*NT spores (10^8^/ml) incubated in agar plates under normoxia and hypoxia conditions. (B) 3D reconstruction of T1-weighted MR images showing spatial propagation of signal from the Gd-spore injection site over time. (C) Quantitative analysis of T1 signal intensity profiles obtained from region of interest (ROI) measurements in corresponding MRI slices, confirming Gd-bacteria proliferation and migration-dependent signal propagation.

*C. novyi-*NT is motile, equipped with peritrichous flagella (flagella distributed all around the cell surface). These enable flagellar-based swimming and swarming motility in liquid or semi-solid environments, respectively. In hypoxic and necrotic tumor cores (rich in cell debris, low oxygen, and soft extracellular matrix), the viscosity is relatively low, supporting flagellar propulsion to be spatially distributed in tumor region. To evaluate whether vegetative Gd-bacterial migration and expansion could be spatially tracked with MRI, 20-mm petri dishes containing 0.1% agar medium were prepared and Gd-*C. novyi*-NT spores were infused in a punched hole in the center of agarose (Fig. 3A). Then, time dependent Gd-*C. novyi*-NT bacterial distribution was monitored using T1-weighted MRI and compared with nonlabeled *C. novyi-*NT spores. After 24 and 72 h of hypoxic incubation, the injected Gd-labeled spores exhibited a marked increase in T1-weighted signal dots, expanding from the initial injection site to outer distant regions across the petri dish. The radial spatial diffusion of T1 contrast from the central injection point suggests that T1-weighted MRI sequences can monitor the active migration of vegetative Gd-*C. novyi*-NT bacteria. These data were markedly different from nonlabeled *C. novyi*-NT spores. Further, under normoxia conditions, the injected Gd-spores initially exhibited a T1 void region at the center with a strong T1 signal in the interface between sample solution and agar. These T1-weighted images were not changed in case of nonproliferative normoxia conditions over 72 h of incubation time. When the T1 signal distributions of Gd-*C. novyi*-NT spores incubated in each condition are converted to threshold images (gray scale over 10), such T1 signal distributions from germinated Gd-*C. novyi*-NT could be clearly visualized and compared in 72 h (Fig. 3B and C). Collectively, our data demonstrate that *C. novyi*-NT spore germination and subsequent bacterial migration can be dynamically monitored over a multi-day window, with detectable MRI signal changes emerging within 24 h and radially expanding over approximately 72 h under hypoxic conditions. These results highlight that a multi-day MRI T1 imaging regimen using Gd-labeled spores and bacteria enables quantitative visualization of the spatiotemporal progression of bacterial therapy within tumors, providing sustained contrast for longitudinal monitoring and assessment.

### In vivo MRI monitoring of hepatic IA infused Gd-spore and Gd-*C. novyi*-NT in orthotropic liver tumor

**Fig. 4.**
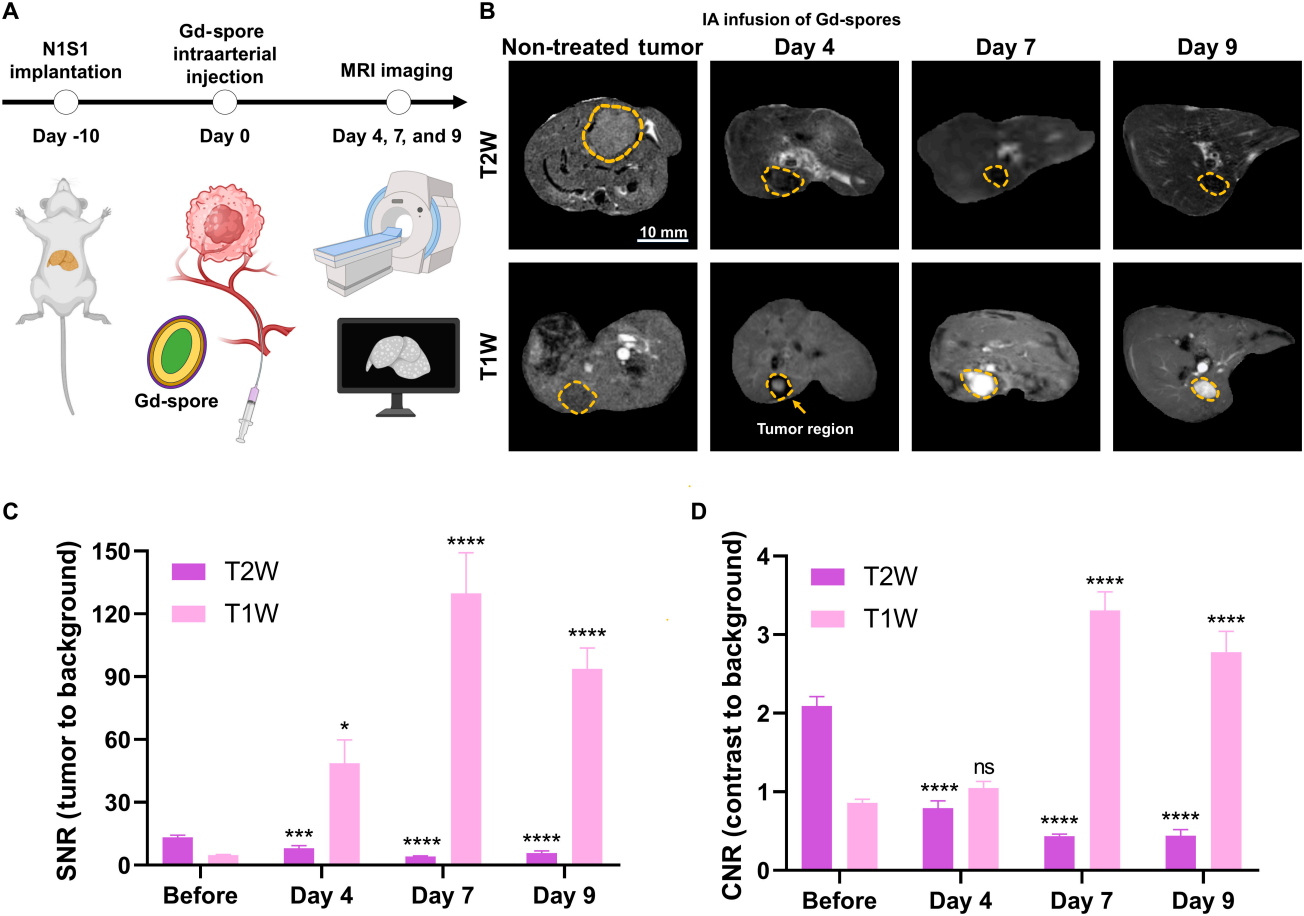
In vivo MRI tracking of Gd-spores in an orthotopic liver tumor model. (A) In vivo experimental schedule for N1S1 tumor implantation, intra-arterial administration of Gd-spores (10^8^/ml, prepared with 10 mM GdCl_3_), and MRI. (B) Representative T1-weighted (T1W) and T2-weighted (T2W) MR images showing dynamic changes in tumor contrast following intra-arterial infusion of Gd-spores. (C) Tumor signal-to-noise ratio (SNR) and (D) contrast-to-noise ratio (CNR) on T1-weighted MR images up to 9 days (*n* = 3). Data are presented as mean ± SD (*P < 0.05, ***P < 0.001, ****P < 0.0001).

Recent advancements in interventional radiology have enabled active research on the IA delivery of *C. novyi*-NT spores, complementing its intravenous and intratumoral delivery methods. In this study, Gd-spores were delivered into the hepatic artery of orthotopic N1S1 tumor-bearing rats. The total Gd exposure under this condition was approximately 0.003 mmol/kg, representing more than a 30-fold reduction relative to a standard single clinical Gd-based contrast agent dose (0.1 mmol/kg). Moreover, systemic Gd exposure is expected to be further minimized by the hepatic IA route of administration [38,39]. Subsequent T2- and T1-weighted MRI scans were conducted to evaluate the Gd-spore delivery, localization, and distribution of Gd-*C. novyi*-NT over a period of up to 9 days (Fig. 4A). Before the IA infusion of Gd-spores, T2-weighted images showed brighter signals of the tumor region compared to the surrounding extrahepatic liver tissue, while T1-weighted images exhibited darker tumor contrast compared to extrahepatic regions (Fig. 4B). After IA infusion of Gd-spores, tumor localized Gd-spores appeared dark contrast in T2-weighted images, and T1 weighted image of tumor showed void signals. It suggests that the IA infusion successfully delivered Gd-spores in the tumor. Those T2 and T1 contrasts were gradually changed from 4 to 9 days post-infusion. Time-dependent changes of T1 signal-to-noise ratio (Fig. 4C) and T1 contrast-to-noise ratio (Fig. 4D) of tumors clearly demonstrated the enhanced T1 contrast effect of Gd-bacteria reaching 120 and 3.3, respectively. This T1 contrast enhancement slightly decreased at 9 days post-IA infusion, but their visibility persisted in whole tumor regions. These results provide crucial evidence that Gd-spores and germinated Gd-*C. novyi*-NT can be effectively tracked and monitored via MRI in vivo, supporting their potential as MR image-guided oncolytic *C. novyi*-NT therapy.

## Conclusion

Noninvasive visualization and tracking of oncolytic bacteria within tumors are highly beneficial for the successful application and development of bacteria cancer therapies. In this study, an MRI visible paramagnetic Gd component was used to label *C. novyi*-NT spores. Electrostatic binding of Gd^3+^ ions to the spore surface formed a clustered Gd layer without compromising spore viability or their capacity to germinate into vegetative bacteria. The stability of Gd labeling on the spore surface and its retention during bacterial proliferation were confirmed by elemental investigation, demonstrating that germinated bacteria effectively retained Gd ions. This Gd retention during bacterial proliferation facilitates MRI of both spore and vegetative bacteria. MRI experiments revealed that Gd-spores infused into the agar phantom generated distinct T2-weighted contrast due to the condensed Gd cluster layer on the spore surface. Under hypoxic, proliferative conditions, this T2-dominant contrast progressively converted to T1-weighted enhancement, enabling noninvasive visualization of spore germination and subsequent bacterial metabolic fission and migration within hypoxic tumor environments. The emergence of T1 contrast during germination therefore provides a robust imaging readout of bacterial activation and enables longitudinal tracking of bacterial migration using T1-weighted MRI. Finally, in vivo studies in orthotopic liver tumor models demonstrated that MRI T2 scanning effectively monitored localized tumoral delivery of Gd-spores following hepatic IA infusion. Sequential longitudinal MRI T1 scans confirmed metabolic proliferation of *C. novyi-*NT bacteria, with T1 contrast at tumor sites persisting for up to 9 days post-infusion. It offers real-time insights into bacterial germination and tumoral distribution. These findings highlight the potential of MRI monitoring of Gd-spores to address key limitations of existing imaging approaches for bacteria cancer therapies. The ability to monitor both infused Gd-spores and metabolically active Gd-bacteria in a proliferation-dependent manner establishes a robust platform for noninvasive, real-time visualization of anaerobic oncolytic bacterial dynamics, supporting the assessment of therapeutic efficacy and safety. Future studies should investigate the integration of this Gd-spore imaging platform with complementary therapeutic modalities, including immunotherapies and local ablation therapies, to further enhance the precision and effectiveness of bacterial cancer treatment.

## Ethical Approval

All experimental procedures were approved and reviewed by the Institutional Animal Care and Use Committee of Northwestern University (IS00003865).

## Data Availability

The data that support the findings of this study are available on request from the corresponding author.
