# Supplementary material for: Metabolic Gadolinium Labeling of Clostridium novyi-Nontoxic for Magnetic Resonance Imaging Visualization of Spores and Germinated Bacteria
Source: Biomater Res. 2026 Feb 10;30:0326. doi: 10.34133/bmr.0326 (PMC12886714; doi:10.34133/bmr.0326)
Supplement: Supplementary 1 — Fig. S1 [file bmr.0326.f1.pdf]

Supplemental Material

Metabolic Gadolinium Labeling of *Clostridium novyi*-Non-Toxic for Magnetic Resonance Imaging Visualization of Spores and Germinated Bacteria

Hyunjun Choi[1†], Jun-Hyeok Han[1†], Sanghee Lee[1], and Dong-Hyun Kim[1,2,3,4*]

[1] Department of Radiology, Feinberg School of Medicine, Northwestern University, Chicago, IL 60611, USA
[2] Department of Biomedical Engineering, McCormick School of Engineering, Evanston, IL 60208, USA
[3] Robert H. Lurie Comprehensive Cancer Center, Chicago, IL 60611, USA
[4] Department of Bioengineering, University of Illinois at Chicago, Chicago, IL 60607, USA

* Address correspondence to: dhkim@northwestern.edu
† These authors contributed equally to this work.

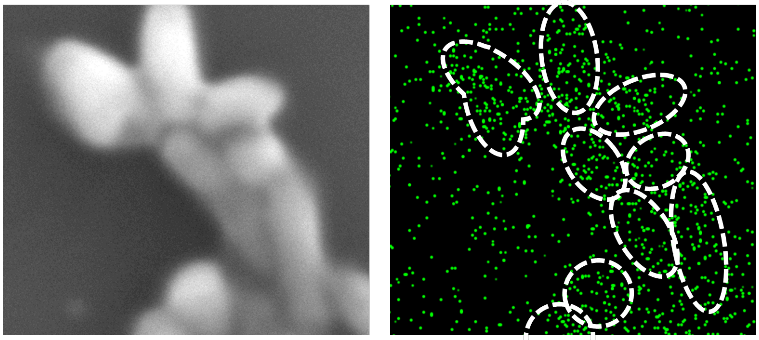

Figure S1. SEM and EDS analysis for Gd elemental mapping (green signal) of Gd-spores (10^8 per mL, prepared with 10 mM $GdCl_3$).
